# Norepinephrine versus phenylephrine infusion for preventing postspinal hypotension during cesarean section for twin pregnancy: a double-blinded randomized controlled clinical trial

**DOI:** 10.1186/s12871-022-01562-3

**Published:** 2022-01-08

**Authors:** Zijun Chen, Jieshu Zhou, Li Wan, Han Huang

**Affiliations:** 1 Department of Anesthesiology & Key Laboratory of Birth Defects and Related Diseases of Women and Children (Sichuan University), Ministry of Education, West China Second University Hospital, Sichuan University, 20#, 3rd Segment, Ren Min Nan Lu, Chengdu, 610041 P.R. China; 2 Department of Anesthesiology, Southwest Hospital, Third Military Medical University (Army Medical University), No.30 Gaotanyan Road, Shapingba district, Chongqing, 400038 China; 3 Department of Obstetrics & Key Laboratory of Birth Defects and Related Diseases of Women and Children (Sichuan University), Ministry of Education, West China Second University Hospital, Sichuan University, Chengdu, 610041 China; 4 Translational Neuroscience Centre, West China Hospital, Sichuan University, Chengdu, 610000 China

## Abstract

**Background:**

Compared with singleton pregnancy, twin gestation is featured by a greater increase in cardiac output. Therefore, norepinephrine might be more suitable than phenylephrine for maintaining blood pressure during cesarean section for twins, as phenylephrine causes reflex bradycardia and a resultant decrease in cardiac output. This study was to determine whether norepinephrine was superior to phenylephrine in maintaining maternal hemodynamics during cesarean section for twins.

**Methods:**

Informed consent was obtained from all the patients before enrollment. In this double-blinded, randomized clinical trial, 100 parturients with twin gestation undergoing cesarean section with spinal anesthesia were randomized to receive prophylactic norepinephrine (3.2 μg/min) or phenylephrine infusion (40 μg/min). The primary outcome was the change of heart rate and blood pressure during the study period. The secondary outcomes were to compare maternal complications, neonatal outcomes, Apgar scores and umbilical blood acid-base status between the two vasopressors.

**Results:**

There was no significant difference observed for the change of heart rate between two vasopressors. The mean standardized area under the curve of heart rate was 78 ± 12 with norepinephrine vs. 74 ± 11 beats/min with phenylephrine (mean difference 4.4, 95%CI − 0.1 to 9.0; *P* = .0567). The mean standardized area under the curve of systolic blood pressure (SBP) was significantly lower in parturients with norepinephrine, as the mean of differences in standardized AUC of SBP was 6 mmHg, with a 95% CI from 2 to 9 mmHg (*P* = .0013). However, requirements of physician interventions for correcting maternal hemodynamical abnormalities (temporary cessation of vasopressor infusion for reactive hypertension, rescuing vasopressor bolus for hypotension and atropine for heart rate less < 50 beats/min) and neonatal outcomes were also not significantly different between two vasopressors.

**Conclusion:**

Infusion of norepinephrine was not associated with less overall decrease in heart rate during cesarean section for twins, compared with phenylephrine.

**Trial registration:**

Chinese Clinical Trial Registry (ChiCTR1900021281).

Compared those with singleton pregnancy, the intensified cardiovascular adaptations with twin pregnancy are characterized by more profound increase in cardiac output (CO) starting from the mid-trimester [1, 2]. As more than 70% of the twin pregnancies were delivered by cesarean section [3, 4], a stable peri-cesarean maternal hemodynamics with well-maintained CO is of vital importance for improving both maternal and neonatal outcomes. Currently, anesthetic management of cesarean section with twins is largely the same as with singleton pregnancy, i.e. spinal anesthesia is preferred over general anesthesia and post-spinal hypotension should be actively managed with vasopressor.

Phenylephrine (PE) is currently the recommended vasopressor of choice for treating/preventing spinal anesthesia -induced hypotension during cesarean section [5]. As a pure α-agonist, PE causes reflex bradycardia which may lead to decrease in maternal CO [6]. Over the past few years, norepinephrine (NE), a potent α-agonist with additional weak β-adrenergic agonist activity, has been introduced into the practice of obstetric anesthesia. A previous study has suggested that the positive chronotropic action of NE counteracts reflex bradycardia caused by pure α-adrenergic activation and NE is therefore associated with greater maternal CO during cesarean section for singleton pregnancy [7]. A higher maternal heart rate (HR) in twin pregnancy contributes substantially to the augmented CO compared to singleton pregnancy. Therefore, NE could be particularly beneficial to parturients with twin pregnancy if it was shown that its use in this specific setting would better preserve maternal HR, as compared to the bradycardic effect of PE. However, comparison between PE and NE during cesarean section for twin pregnancy is lacking as twin pregnancy was often excluded in previous studies in which different vasopressors were compared [7–10].

The primary objective of this randomized, double-blinded study was to compare the changes in heart rate and blood pressure following prophylactic intravenous infusions of norepinephrine or phenylephrine in women with twin pregnancies undergoing cesarean section under spinal anesthesia. The secondary objectives were to compare maternal complications, neonatal outcomes, Apgar scores and umbilical blood acid-base status between the two vasopressors.

## Methods

**Fig. 1:**
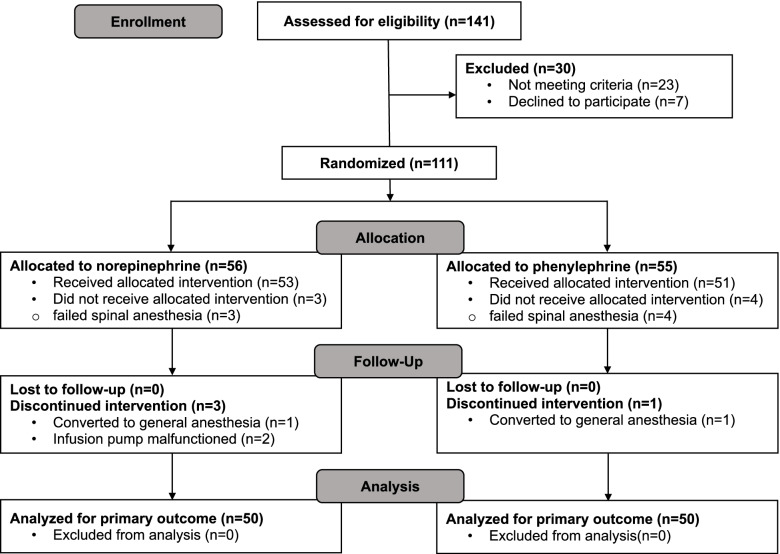
CONSORT flow diagram showing patient recruitment and flow

This study was reviewed and approved by ethics committee (full name of the committee: China Ethics Committee of Registering Clinical Trials; reference No. ChiECRCT-20,190,007). Study protocol was registered in the Chinese Clinical Trial Registry prior to patient enrollment (Registration no. ChiCTR1900021281, Principal investigator: Dr. Jieshu Zhou, Date of registration: 11 February 2019, weblink for the online registration: http://www.chictr.org.cn/showproj.aspx?proj=35234). Informed consent was obtained from all patients before enrollment. This manuscript adheres to the applicable CONSORT guidelines (Fig. 1). The authors assert that all procedures contributing to this work comply with the ethical standards of the relevant national and institutional committees on human experimentation and with the Helsinki Declaration of 1975, as revised in 2008.

Parturients with twin pregnancies were screened according to the following inclusion criteria: American Society of Anesthesiologists physical status I-III, gestational age between 32 and 38^+ 6^ weeks, going to receive cesarean section with spinal anesthesia, and estimated body weight ≥ 1500 g for both fetuses. Parturients with cardiovascular disease, hypertensive disorder, onset of labor, contraindications to spinal anesthesia or known severe fetal abnormality would be excluded. Patients who underwent cesarean section due to non-reassuring fetal status or received general anesthesia for the cesarean section would also be excluded.

Upon entering the operating room, patients were positioned on the operating table in supine position tilted to the left for 15° to avoid possible aortocaval compression as per our department protocol. Patients were also positioned in this left tilt position during the cesarean procedure. Standard monitoring was applied, including 5-lead electrocardiography, non-invasive blood pressure, and pulse oximetry. Blood pressure and heart rate were measured and recorded at 1-min interval till the difference between 3 consecutive measurements was less than 10%. The mean value of these 3 measurements were recorded as baseline value.

Two 18-G intravenous catheters were inserted into forearm veins while no fluid preload was given. Then, patients were positioned in left lateral position while spinal anesthesia was performed. 2.5 ml of 0.5% plain isobaric bupivacaine were injected intrathecally at L3-L4 or L2-L3 intervertebral space via a 25-G pencil-point spinal needle. Intrathecal administration of opioids was spared in this study.

Sensory blockade level was assessed by pinprick and surgery would not begin unless the sensory block reached T6 level. Patients with inadequate sensory blockade would receive general anesthesia for cesarean delivery, who would then be excluded from this study.

Infusion of study drug was initiated when spinal injection started. Before starting the study, a simple randomization sequence for 100 codes divided into two equal-sized groups was generated using SPSS software (version 20.0; SPSS Inc., Chicago, IL). One code for each patient was placed into a sealed, opaque, sequentially numbered envelope by one investigator (Z.C.), who was not involved in patient management or data collection. After epidural space was reached, another investigator (L.W.), who was not involved in subsequent patient care or assessment either, opened the topmost of these consecutively numbered the envelops. According to the code contained in the envelops, patients were randomly allocated to receive PE or NE infusion. All the PE and NE solutions were prepared in identical 20 ml syringes by J.Z., who was not involved in subsequent patient care or assessment either. All the syringes were labelled as “study drug”, therefore it was impossible for the anesthesiologists involved in patient care to identify whether it contained 8 μg/ml NE (norepinephrine bitartrate injection, 2 mg/ml; Grandpharma Co. Ltd., Wuhan, China) or 100 μg/ml PE (phenylephrine hydrochloride injection, 10 mg/ml; Harvest Pharmaceutical Co. Ltd., Shanghai, China). The infusion rate was 24 ml/hour for both vasopressors by using an infusion pump (BeneFusion SP3D, Mindray Co. Ltd., Shenzhen, China), corresponding to 3.2 μg/min for NE and 40 μg/min for PE. In our department, continuous PE infusion at 40 μg/min is the standard protocol for post-spinal hypotension prophylaxis during cesarean delivery. The infusion rate of NE was determined based on the estimated potency of 12.5:1 for NE:PE, according to the previously reported potency from 11.3 [11] to 13.1:1 [12] for NE:PE. Study drug was flushed into the blood stream by carrier lactated Ringer’s solution infused at the rate of 1 ml · kg^− 1^ · hour^− 1^. Infusion of study drug was stopped when the second twin was delivered, and the study was then terminated. Both the patients and the anesthesiologists were blinded for the patients’ assignments. Ringer’s solution co-loading via another peripheral venous catheter was initiated as infusion of the study drug began. The infusion bags were suspended about 1.5 m above the parturient’s right atrium with the clamp fully opened till the study ended. Then, the fluid infusion rate was adjusted at the discretion of the anesthesiologist.

Blood pressure was measured and recorded every minute till delivery of the second twin. Hypotension, defined as a systolic blood pressure (SBP) < 90 mmHg or 80% of the baseline SBP, was rescued by 1-ml bolus of the study drug, i.e. NE 8 μg for parturients with NE infusion and PE 100 μg for parturients with PE. These boluses were prepared beforehand in 10-ml syringes labelled as “rescuing drug”. Hypertension, defined as a SBP > 140 mmHg or 120% of the baseline, was managed by temporarily stopping study drug infusion, which would be restarted as soon as SBP was within the normal range. HR was monitored continuously and recorded at 1-min interval. Bradycardia was defined as a heart rate < 60 beats/min and 0.5 mg atropine would be given when heart rate further decreased below 50 beats/min. Oxygen 5 L/min was given by a facemask during the study period.

Apgar scores were assessed by midwives 1, 5 and 10 min after delivery, who were unaware of the patients’ assignment. Umbilical arterial (UA) and venous blood (UV) were sampled from a double-clamped segment of umbilical cord by using Pulset arterial 3 ml blood gas syringes (Westmed, AZ85706, USA). Within 20 min after clamping, umbilical blood gas was analyzed with the bedside epoc® Blood Analysis System (Siemens Healthcare GmbH, Erlangen, Germany) in the operating room.

The primary outcome was the change in heart rate and blood pressure during the study period, from intrathecal injection to delivery of the second twin, which was calculated as the standard area under the curve of heart rate and systolic blood pressure (please find below, in the section of statistical analysis). The secondary outcomes included the incidences of hypotension, hypertension, bradycardia, nausea and vomiting, umbilical blood gas status, Apgar scores, both maternal and neonatal length of post-operative stays, and rates of maternal and neonatal intensive care unit (NICU) admission.

### Statistical analysis

Continuous data such as pH values were assessed for normal distribution using Kolmogonov-Smirnov test and were then compared using the Student’s t-test or Mann-Whitney U test, as appropriate. Categorical data such as the incidence of bradycardia were analyzed using chi-square test. A survival analysis with log-rank test was used to compare the requirement of physician interventions for correcting maternal hemodynamic abnormalities (including injection of rescuing bolus of vasopressors or atropine and temporary cessation of vasopressor infusion). The area under the curves (AUC) for SBP and heart rate plotted against time were calculated using the trapezium rule [7]. As the surgical time varied among parturients, a variable number for heart rate and blood pressure measurements were recorded for each parturient. Each AUC value was divided by the number of data points recorded to give the standardized AUC for each patient, which were compared between two vasopressors using the Student’s t-test. Analysis was performed with Prism (version 8.4.3, GraphPad Software, San Diego, California USA). A *p* value < 0.05 was considered statistically significant.

The sample size was calculated based on our unpublished preliminary data, in which parturients with twin pregnancy received NE or PE infusion following spinal anesthesia. The means of standardized AUC for heart rate were 79 ± 12 and 72 ± 8 beats/min for NE and PE, respectively. A minimal sample size of 46 per group was required to detect a significant difference (10% of the baseline heart rate) between two groups, with 90% power and a two-sided type I error of 0.05. Considering the potential withdraw of consent or loss of follow-up, a final sample size of 50 per group was determined.

## Results

This study was conducted in West China Second University Hospital, Chengdu, China, from November 2019 to April 2020. The CONSORT recruitment diagram is showed in Fig. 1. Totally 141 parturients with twin pregnancy were screened for eligibility and data were analyzed from 50 patients in each group.

**Fig. 2:**
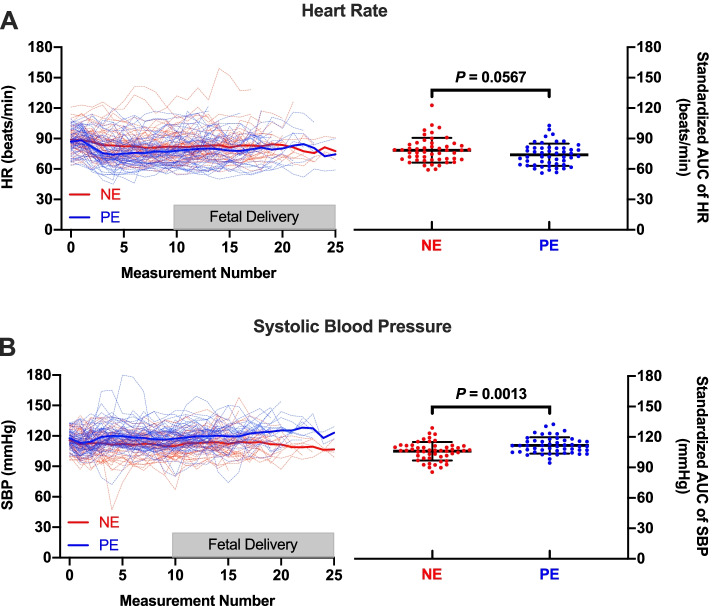
Serial changes in heart rate (**A**) and systolic blood pressure (**B**) following norepinephrine (lines in red) or phenylephrine (lines in blue) infusion. Compared with phenylephrine, infusion of norepinephrine is not associated with less decrease in heart rate (*p* = 0.0567) but with a lower systolic blood pressure (*P* = 0.0013) over the study period. On the left side, dotted lines indicate individual measurements while solid lines indicate the mean values. The shaded area shows the time period within which the newborns are delivered. One the right side, individual data are presented as scattered dots and bars indicate mean and standard deviation. (AUC = Area Under the Curve, HR = Heart Rate and SBP = Systolic Blood Pressure)

Patients’ baselines and surgical characteristics were similar between two groups (Table 1). No patient in our study reported any discomfort due to incomplete blockade or required any supplementary intravenous analgesics or anesthetics during the cesarean procedure. Infusion of NE did not lead to less decrease in heart rate following spinal anesthesia in patients with twin pregnancy and there was no inter-group difference in heart rate over the study period (Fig. 2A). The mean standardized AUC of heart rate was 78 ± 12 and 74 ± 11 beats/min with NE and PE, respectively (mean of difference 4.4 with a 95% confidence interval (CI) - 0.1 to 9.0; *P* = .0567). The incidence of bradycardia, i.e. heart rate < 60 beats/min, was 6% (3/50) in patients receiving NE and 30% (15/50) in patients with PE (odds ratios [OR] with NE = 0.15; 95%CI, 0.04 to 0.56; *P* = .002), as showed in Table 2. Among these patients with bradycardia, only three from group PE met the criteria for atropine administration, i.e. heart rate < 50 beats/min (3/50, 6% versus 0/50, 0%, OR with NE = 0; 95%CI, 0 to 1.11; *P =* .242). And 86.7% (13/15) of the bradycardia episodes in group PE occurred before surgical skin preparation followed by a rapid and spontaneous recovery and only two patients had heart rate less than 60 beats/min when the neonates were delivered.

**Table 1:** Baseline and Surgical Characteristics

	NE (*n* = 50)	PE (*n* = 50)	*P* value
Age, year	31.6 (4.3)	31.5 (4.2)	.400
Weight, kg	72.6 (8.0)	71.0 (11.2)	.415
Height, cm	161 (5.3)	160 (4.7)	.464
Body mass index, kg.m^− 2^	28.0 (2.3)	27.6 (3.5)	.505
Gestation at delivery, week			.913
32^+ 0^ to 34^+ 6^	8 (16%)	7 (14%)	
35^+ 0^ to 36^+ 6^	21 (42%)	23 (46%)	
37^+ 0^ to 38^+ 6^	21 (42%)	20 (40%)	
Parity	1 (0–2)	1 (0–2)	.437
Pregnancy complications ^*a*^	23 (46%)	18 (36%)	.309
Block height, dermatome	6 (4–6)	6 (4–6)	.060
Baseline systolic blood pressure, mmHg	116 (10)	118 (8)	.250
Baseline heart rate, beats/min	89 (12)	87 (12)	.419
Time intervals, second			
Spinal injection to skin incision	1032 (877–1215)	1069 (750–1261)	.907
Skin incision to delivery of first twin	247 (204–345)	230 (184–306)	.272
Interval between two deliveries	72 (54–108)	63 (43–105)	.244
Blood Loss, ml	539 (156)	551 (203)	.741
Nausea and vomiting	2 (4%)	1 (2%)	1.000
Postoperative length of stay, day	3 (3–4)	3 (3–4)	.917

Data reported as median mean (standard deviation), (interquartile range), or number (proportion)

^*a*^ Pregnancy complications include hypothyroidism, intrahepatic cholestasis of pregnancy and gestational diabetes

Abbreviations: *NE* Norepinephrine; *PE* Phenylephrine

**Table 2:** Incidence of Maternal Hemodynamic Abnormalities

	NE (***n*** = 50)	PE (***n*** = 50)	OR with NE	***P*** value
**Bradycardia**^*a*^	3 (6%)	15 (30%)	0.15 (0.04, 0.56)	.002
**Atropine rescue**^*b*^	0 (0%)	3 (6%)	0.00 (0.00, 1.11)	.242
**Hypotension**	12 (24%)	3 (6%)	4.95 (1.30, 18.81)	.012
**Hypertension**	6 (12%)	13 (26%)	0.39 (0.13, 1.12)	.074

Data reported as number (proportion) or OR [95% CI]

^*a*^Bradycardia is defined as heart rate is less than 60 beats/min

^*b*^Atropine is administrated when heart rate is less than 50 beats/min

Abbreviations: *NE* Norepinephrine; *OR* Odds Ratio; *PE* Phenylephrine

**Fig. 3:**
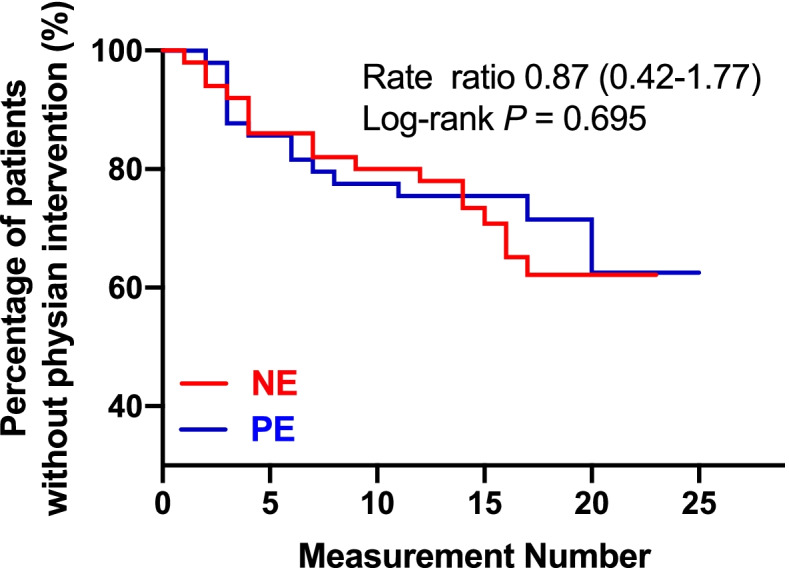
Requirement of physician intervention for correcting maternal hemodynamic abnormalities following norepinephrine or phenylephrine infusion

Patients receiving NE had significantly lower blood pressure than those receiving PE during the study period, as revealed in Fig. 2B. However, the mean of differences in standardized AUC of SBP was 6 mmHg, with a 95% CI from 2 to 9 mmHg (*P* = .0013). The incidence of hypotension was 24% (12/50) with NE and 6% (3/50) with PE (OR with NE = 4.95; 95%CI, 1.30–18.81; *P* = .012). None of the hypotensive patients required more than one bolus of rescuing vasopressor to restore blood pressure. As rescuing bolus was used more frequently in patients with NE, the total volume of vasopressor was greater in group NE: 9.9 ± 2.3 versus 8.5 ± 2.9 mL with mean difference of 1.45; 95%CI, 0.42 to 2.48 (*P* = .006). Hypertension occurred in 6 patients receiving NE (12%, 6/50) and in 13 patients receiving PE (26%, 13/50), OR with NE = 0.39; 95%CI, 0.13–1.12, (*P* = .074). Taking all the above-mentioned maternal hemodynamic abnormalities into consideration (including atropine for heart rate < 50 beats/min, bolus of rescuing vasopressors for hypotension and temporary cessation of study drug infusion for reactive hypertension), there was no difference in requirements of physician interventions between the two groups (Fig. 3).

No newborn had Apgar’s score less than 8 or unplanned NICU admission in either group. As showed in Table 3, there was no difference in neonatal outcomes between the two groups, except that neonates in group NE had a slightly greater body weight. Due to the equipment failure and difficulties in collecting blood, umbilical arterial (UA) blood gas analysis was missed in 13 and 11 neonates in group NE and PE, respectively; umbilical venous (UV) blood gas analysis was missed in 9 neonates in each group. No UV pH value was less than 7.20 in either group while UA pH < 7.20 was found in 2.3% (2/87) of newborns from group NE and 1.1% (1/89) of newborns from group PE (OR with NE = 2.05; 95%CI, 0.24–30.31, *P* = .619). There was no difference in umbilical blood gas status between the two groups, except that neonates from group NE had a slightly higher glucose level, in both UA blood (2.92 ± 0.57 versus 2.71 ± 0.56 mmol/L, *P* = .015) and UV blood (3.39 ± 0.57 versus 3.18 ± 0.67 mmol/L, *P* = .020).

**Table 3:** Neonatal Outcomes Compared between norepinephrine and phenylephrine

	NE	PE	95% CI ^***a***^	***P*** value
	(***n*** = 100)	(***n*** = 100)		
**Birthweight,** g	2493 (388)	2389 (331)	104 (3.3, 204.5)	.043
< 2500	47 (47%)	60 (60%)	0.59 (0.34, 1.04)	.065
< 2000	14 (14%)	10 (10%)	1.47 (0.62, 3.48)	.384
**Height,** cm	46 (2.5)	46 (2.3)	0.3 (−0.37, 0.97)	.379
**Sex**			1.33 (0.76, 2.32)	.320
Female	42 (42%)	49 (49%)		
Male	58 (58%)	51 (51%)		
**Apgar score**				
1 min	10 (8–10)	10 (8–10)	0.00 (0.00, 0.00)	.764
5 min	10 (9–10)	10 (9–10)	0.00 (0.00, 0.00)	.562
10 min	10 (9–10)	10 (9–10)	0.00 (0.00, 0.00)	.156
**NICU Admission**	27 (27%)	30 (30%)	0.86 (0.47, 1.60)	.638
**UA blood gas**	***n*** **= 87**	***n*** **= 89**		
pH	7.292 (0.036)	7.295 (0.035)	−0.003 (−0.014, 0.007)	.538
PCO_2_, mmHg	48.66 (5.39)	48.70 (6.38)	−0.03 (−1.79, 1.72)	.969
PO_2_, mmHg	19.74 (4.79)	18.36 (5.123)	1.38 (−0.10, 2.86)	.067
BE, mmol/l	−3.09 (1.99)	−2.93 (1.91)	−0.17 (− 0.75, 0.41)	.57
SO_2_, %	26.32 (10.64)	23.90 (11.97)	2.42 (−0.95, 5.79)	.158
Glu, mmol/l	2.92 (0.57)	2.71 (0.56)	0.21 (0.04, 0.38)	.015
Lac, mmol/l	2.83 (0.70)	2.76 (0.67)	0.06 (−0.14, 0.27)	.544
**UV blood gas**	***n*** **= 91**	**n = 91**		
pH	7.338 (0.031)	7.334 (.029)	0.005 (−0.004, 0.014)	.263
PCO_2_, mmHg	41.39 (5.33)	42.67 (4.81)	−1.29 (−2.77, 0.20)	.089
PO_2_, mmHg	28.49 (8.09)	27.70 (8.53)	0.79 (−1.64, 3.22)	.521
BE; mmol/l	−3.66 (1.81)	−3.23 (1.89)	−0.43 (−0.97, 0.12)	.122
SO_2_, %	48.02 (16.47)	45.76 (16.80)	2.26 (−2.61, 7.13)	.361
Glu, mmol/l	3.39 (0.57)	3.18 (0.67)	0.22 (0.03, 0.40)	.020
Lac, mmol/l	2.43 (0.57)	2.41 (0.70)	0.02 (−0.16, 0.21)	.796

Data reported as mean (standard deviation), median (range), or number (proportion)

^***a***^95% CI is 95% confidence interval of difference or odds ratio

Abbreviations: *BE* Base excess; *Glu* Glucose; *Lac* Lactate; *NE* Norepinephrine; *NICU* Neonatal intensive care unit; *PE* Phenylephrine; *UA* Umbilical artery; *UV* Umbilical vein

## Discussion

Our study shows that infusion of NE at a fixed rate of 3.2 mcg/min failed to provide less overall decrease in HR compared to PE infused at 40 mcg/min during caesarean delivery for twin pregnancy performed under spinal anesthesia (78 ± 12 vs. 74 ± 11 beats/min, mean difference 4.4; *P* = 0.057); nevertheless, incidence of bradycardia was significantly lower with NE (6% vs. 30%, *P* = 0.002). In addition, with these vasopressor infusion settings, there was a higher incidence of hypotension in the NE group (24% vs. 6%, *P* = 0.01). Overall, requirements of physician interventions to correcting maternal hemodynamic abnormalities (i.e. atropine for heart rate less than 50 beats/min, rescuing vasopressor bolus for hypotension and temporary cessation of vasopressor infusion for reactive hypertension) were similar with these two vasopressor infusion regimen. There was no difference in fetal outcomes either.

Most parturients with twin pregnancy are delivered by cesarean section [3, 4], and their anesthetic management is largely the same as for singleton pregnancy [13]. From this point of view, PE-induced bradycardia and resultant decrease in cardiac output (CO) [14] could be a particular concern during cesarean section for twin pregnancy, as a higher heart rate contributes substantially to the augmented CO developed in twin pregnancy [2].

In our study, NE failed to produce a greater overall HR after spinal anesthesia during cesarean section for twins, although this nearly achieved statistical significance (*P* = 0.0567). In addition, the incidence of bradycardia was significantly higher in patients receiving PE, although the requirements of atropine were not different between the two vasopressors. Nevertheless, most of the bradycardia episodes (13/15) occurred before skin incision and spontaneously recovered afterwards. The similar umbilical blood gas status and fetal outcomes we found between NE and PE is furthermore reassuring. In a recent study recruiting more than 600 parturients with singleton pregnancy, it was also found that umbilical arterial pH was similar between NE and PE, although PE was associated with higher incidence of bradycardia [15]. But other studies have reported better umbilical arterial base excess with NE, versus PE [16, 17], so further studies will still be needed to clarify this issue.

The infusion rate of NE was determined based on the estimated potency of 11.3 [11] to 13.1:1 [12] for NE: PE, as infusion rate of 40 μg/min for PE is the standard protocol for post-spinal hypotension prophylaxis in our department. In our study, infusion of NE at 3.2 μg/min corresponds to a weight-adjusted infusion rate of 0.044 μg · kg^− 1^ · min^− 1^ (ranging from 0.034 to 0.056 μg · kg^− 1^ · min^− 1^). In two recently published papers, which were not available when our study was designed, the ED_95_ of NE, NE bitartrate to be more specific, for maintaining blood pressure for cesarean delivery after spinal anesthesia was estimated to be 0.07 [18] to 0.08 [19] μg · kg^− 1^ · min^− 1^. And the incidence of hypotension with NE infusion at the rate of 0.05 μg · kg^− 1^ · min^− 1^ was as high as 30% [19] or even 47.4% [18] in these dose-finding studies. All these data suggested that NE infusion at 3.2 μg/min was too low for post-spinal hypotension prevention. However, these data were both derived from singleton pregnancy and were with wide confidence intervals, so one should be cautious not to directly extrapolate these data to twin pregnancy. Equipotency of both vasopressors need to be confirmed in women with twin pregnancy and maternal/fetal outcomes should be compared with these equipotent regimens in future studies. When comparing NE dosages between different studies, care must be taken to identify which NE preparation was used (i.e., brand name and manufacturer), especially if the NE concentration is expressed as NE base or NE bitartrate, since base is twice as potent as bitartrate formulation (i.e., 5 mcg/ml of NA base = 10 mcg/ml of NA bitartrate [20].

In this study, we included a wide spectrum of gestational ages, which is not uncommon in studies of twin pregnancy [13]. Although gestational age could impact hypotension rates during caesarean delivery, the equal randomization of gestational ages between groups in our study suggest that this had little impact on between group differences (Table 1).

There are several limitations in our study. First, vasopressors were infused at a fixed rate for all the patients, instead of manually adjusted infusion [16] or closed-loop feedback computer-controlled infusion [7]. Further study is warranted to define the best infusion protocol. Another limitation is that we did not measure maternal cardiac output directly, but measured heart rate which is regarded as the “best surrogate indicator of cardiac output during cesarean delivery” [21]. The major reason for the missing of cardiac output measurement is that there was a hardware failure of our bioimpedance-based non-invasive CO monitoring device just before the study began. Moreover, there has been concerns about the accuracy of non-invasive CO monitors especially when used in pregnant women [22]. Therefore, the heart rate was considered as a surrogate. Finally, PE and NE were not compared at the equipotency, which contributed to the imbalance in blood pressure control between the two groups. As we mentioned earlier, equipotency of both vasopressors need to be confirmed in women with twin pregnancy and maternal/fetal outcomes should be compared with these equipotent regimens in future studies.

In conclusion, in parturients with twin pregnancy undergoing cesarean delivery under spinal anesthesia, infusion of NE at the rate of 3.2 mcg/min failed to provide less overall decrease in HR, compared to PE infused at 40 mcg/min, although there was a significant decrease in incidence of bradycardia. The requirements of physician intervention to correcting maternal hemodynamic abnormalities and the fetal outcomes were also similar with these two vasopressors infusion regimen.

## Data Availability

All data generated or analyzed during this study are included in this published article.
